# 4148 Thrombocytopenia and whole blood transfusion in children with severe falciparum malaria

**DOI:** 10.1017/cts.2020.153

**Published:** 2020-07-29

**Authors:** Matthew M. Ippolito, Jean-Bertin Kabuya, Manuela Hauser, Benjamin Kussin-Shoptaw, Austin Peer, Marco Ruegg, Albert Baschong, Thomas Louis, William Moss

**Affiliations:** 1 Johns Hopkins University School of Medicine; 2 Tropical Diseases Research Centre; 3 University Children’s Hospital Zurich; 4 Johns Hopkins University School of Public Health; 5 University Hospital Basel

## Abstract

OBJECTIVES/GOALS: Severe malarial anemia due to *Plasmodium falciparum* is often accompanied by thrombocytopenia. Treatment includes transfusion of whole blood, which contains erythrocytes, platelets, and other blood components. The objective of the study was to assess the effect of whole blood transfusion on survival in children with severe falciparum malaria and to examine the potential interaction of thrombocytopenia with malaria mortality and transfusion response. METHODS/STUDY POPULATION: We analyzed a retrospective cohort of 842 hospitalized children in Zambia with severe malarial anemia (703 transfused, 139 not transfused due to stock-out or other reason). Severe malarial anemia was defined as a positive rapid diagnostic test or blood smear in combination with an admission hemoglobin concentration ≤5 g/dL. RESULTS/ANTICIPATED RESULTS: Mortality was 13% (94/703) in the transfused group and 24% (34/139) in the non-transfused group. Kaplan-Meier survival estimates stratified by transfusion status and thrombocytopenia (150,000/μL threshold) showed increased mortality in children with thrombocytopenia who did not undergo transfusion, with no differences in mortality among the other transfused and non-transfused groups (log-rank test P = 0.0001). Effect modification analysis by Cox proportional hazards regression adjusted for age, sex, hemoglobin concentration, blood group type, and eosinophilia showed a significant interaction between platelet count and transfusion status (P = 0.028). Children with thrombocytopenia who were transfused and died had little or no post-transfusion increase in platelets, in contrast to those who survived. Freshness of transfused whole blood, construed from expiration dates, correlated with greater platelet recovery and improved survival. DISCUSSION/SIGNIFICANCE OF IMPACT: The role of platelets in malaria pathophysiology is complex and incompletely understood; prior studies describe preferential binding of platelets to parasitized erythrocytes and direct parasitocidal activity, whereas others detailed deleterious effects in malaria involving the central nervous system vasculature. These findings point to a potential clinical role for platelet-directed transfusion strategies to improve survival in children with severe falciparum malaria, which should be further assessed in randomized interventional studies.

